# Dog Breed Differences in Visual Communication with Humans

**DOI:** 10.1371/journal.pone.0164760

**Published:** 2016-10-13

**Authors:** Akitsugu Konno, Teresa Romero, Miho Inoue-Murayama, Atsuko Saito, Toshikazu Hasegawa

**Affiliations:** 1 Japan Society for the Promotion of Science, Kojimachi 5-3-1, Chiyoda, Tokyo, Japan; 2 Department of Cognitive and Behavioral Science, The University of Tokyo, Komaba 3-8-1, Meguro, Tokyo, Japan; 3 Wildlife Research Center, Kyoto University, Tanaka Sekiden cho 2–24, Sakyo, Kyoto, Japan; University of Portsmouth, UNITED KINGDOM

## Abstract

Domestic dogs (*Canis familiaris*) have developed a close relationship with humans through the process of domestication. In human-dog interactions, eye contact is a key element of relationship initiation and maintenance. Previous studies have suggested that canine ability to produce human-directed communicative signals is influenced by domestication history, from wolves to dogs, as well as by recent breed selection for particular working purposes. To test the genetic basis for such abilities in purebred dogs, we examined gazing behavior towards humans using two types of behavioral experiments: the ‘visual contact task’ and the ‘unsolvable task’. A total of 125 dogs participated in the study. Based on the genetic relatedness among breeds subjects were classified into five breed groups: Ancient, Herding, Hunting, Retriever-Mastiff and Working). We found that it took longer time for Ancient breeds to make an eye-contact with humans, and that they gazed at humans for shorter periods of time than any other breed group in the unsolvable situation. Our findings suggest that spontaneous gaze behavior towards humans is associated with genetic similarity to wolves rather than with recent selective pressure to create particular working breeds.

## Introduction

Domestic dogs (*Canis familiaris*) have been living close to humans (*Homo sapiens*) for at least 15,000 to 50,000 years, a relationship that probably came about through multiple domestication events [1–5]. Dogs are currently thought to be one of the best models for understanding cognitive skills in cross-species communication [6–9], and a number of studies have focused on the ability of dogs to comprehend and respond to various types of human communicative signals (e.g., [6,8,10,11]). For instance, it is known that dogs are able to process many types of human gestures including pointing, bowing, nodding, head turning and gazing as cues for finding the location of hidden food [11].

It has been suggested that the skills required by dogs to interact with humans were acquired through the process of domestication (e.g., [8,12]). Comparative studies of dogs and their closest living relative, the wolf (*Canis lups*), have shown that hand-reared wolves are less responsive to human social cues and less prone to showing human-directed gaze signals than domestic dogs [9,13–16]. Studies of captive silver foxes (*Vulpes vulpes*) also offer an insight into behavioral modification in domesticated canids: fox strains selected for tameness are more sensitive to human pointing gestures than non-selected individuals [17–19]. These findings suggest that an early divergence from wolves, possibly by artificial selection for tameness, may have had a significant impact on modern dog’s communicative abilities [8].

However, modern dog’s behavior has been modified not only during the early stages of domestication, but also during a later period of breed creation [8,20]. Most modern purebred dogs were established in Europe through an intensive selective breeding process for preferred behavioral/phenotypic traits about 200 to 500 years ago. At present, the current number of breeds worldwide has reached almost 400 [21–25]. It is important to note that a subgroup of modern purebred dogs, referred to as ‘Ancient’, ‘Primitive’, or ‘Basal’ breeds, have experienced lower selective pressure. In general, these breeds have kept a high genetic diversity and possess similar genetic components to wolves due to their earlier isolation [1,21,22,26,27]. Besides this important characteristic, Ancient breeds have not always been included in studies examining the evolution of dog communicative skills (but see [28–30]). To evaluate the impact of artificial selection on dog behavior, the diversity of modern purebred dogs, including Ancient breeds, should be considered.

Examining dog breed differences in behavior could shed light on the acquisition process of their extraordinary abilities to communicate with humans. Thus, one could hypothesize that if dog communicative skills developed during the period of the early split between wolves and dogs, then more wolf-like dog breeds (i.e., Ancient breeds) may be less skilled at communicating with humans [30,31]. This ‘wolf remnant’ hypothesis has been supported by results from recent studies of two wild dog breeds belonging to the Ancient group (i.e., dingo and New Guinea singing dog). For instance, while dingoes are able to follow several human gestures in order to locate hidden food, including tapping, pointing and standing behind the container, they fail to respond to human gaze cues [31]. Since other breeds are generally successful in using human gaze signals, Ancient breed performance appears to lie somewhere between that of wolves and other dogs [30,31]. This suggests that a dog’s ability to comprehend human gestures is associated with genetic closeness to wolves.

An alternative hypothesis for the source of modern dogs’ behavior holds that intensive selective pressure for particular working purposes has significantly changed their ability to communicate and interact with humans [28,30]. A recent study has shown that breeds selected to work in close cooperation with humans (e.g., Shepherds and Huskies) are more effective in using human pointing gestures than breeds not selected to work with humans (e.g., Basenji and Toy Poodle) [30]. The authors emphasized that working breeds outperform non-working ones in this regard independently of being genetically wolf-like. Furthermore, breeds selected to work in close cooperation and in visual contact with humans (e.g., sheepdogs and gun dogs) have been proven to be more skilled at reading human pointing gestures than other working breeds (e.g., hounds and earth dogs) [28]. These findings are in line with the ‘working purpose’ hypothesis, which states that breed selection for specific types of cooperative activity with human partners has contributed a large degree to the communicative behavior of dogs.

Compared to the skills used in responding to human-given cues, dog’s ability to produce communicative signals towards humans has received much less attention and only recently a few studies have begun to focus on this topic. It has been shown that hand-reared wolves are less inclined to show gaze signals towards humans in a problem-solving situation than domestic dogs [16]. Based on this finding, Passalacqua *et al*. [29] examined the potential effect of breed groups (Primitive, Hunting/Herding and Molossoid groups) on spontaneous gazing behavior towards humans when the dog also faced a problem-solving situation. Contrary to the authors’ prediction that Primitive breeds would be less prone to showing human-directed gazing behavior (i.e., the ‘wolf remnant’ hypothesis), Primitive and Molossoid groups showed similar gazing behavior while both groups were outranked by Hunting/Herding breeds. On the other hand, studies evaluating gaze responses in a direct human-to-dog feeding interaction (with food in sight but out of reach) found significant breed differences in human-directed gazing behavior (e.g., [32][33]). For instance, in one study, Retrievers (a hunting breed specialized in retrieving prey) spontaneously gazed at humans for longer periods of time than German Shepherds (a herding or livestock protecting breed) or Poodles (a companion breed) [34]. Although these results seem to support the ‘working purpose’ hypothesis, the limited number of breeds and working types included in these studies does not allow any firm conclusion to be drawn.

In summary, previous data from studies on breed differences in communicative behavior provides partial support for both the ‘wolf remnant’ and the ‘working purpose’ hypotheses, and hence it is not clear whether genetic similarity to wolves or to working types has a greater influence on modern dogs’ abilities to communicate with humans. Given that spontaneous gazing at humans can facilitate the initiation and maintenance of dog-human communication and bonding [16,32,33,35], further research examining how the domestication process has contributed to modern dog’s use of gazing behavior towards human is warranted.

The aim of the present study was to estimate the influence of selective pressures on the ability of dogs to spontaneously produce communicative signals such as eye contact and gazing towards humans. In particular, we predict that if the genetic remnant of wolves has a significant influence on modern dog’s behavior then Ancient breeds would show less human-directed gazing behavior than other purebred dogs. In contrast, if selection for some specific working purposes had a significant influence in the development of dog communicative abilities, then particular working breed groups would display a greater capacity for human-directed gazing behavior. Although dog’s genetic similarity with wolves and its selection history for working purposes has been closely intertwined, the recently published data on the genetic clustering of dog breeds brings a tentative solution to estimate indirect impact of genetic factors on dog’s behavior [21].

To advance the current scarcity of data on the production of communicative signals by dogs, the present study aims to address some of the methodological issues of previous studies. Firstly, we tested a wide range of 26 pure breeds including major modern pure breeds as well as ancient breeds. These breeds were further classified into broader breed groups (i.e., Ancient, Herding, Hunting, Retriever-Mastiff and Working) that cluster genetically clusters according to recent genomic analysis [21]. Grouping breeds in this way is important in order to estimate the possible effect of selective pressures that may be shared by more than one breed, as well as to compare inter-breed variation in ability to exchange communicative signals with humans. Secondly, we used two different experimental paradigms to draw spontaneous gaze responses towards humans when requesting out-of-reach food rewards: the ‘unsolvable task’ and the ‘visual contact task’. The use of multiple behavioral tasks allows us to examine whether each breed group has a consistent behavioral pattern for sending communicative signals to humans independently of the situation or task. Finally, for comparative purposes, we will also analyze our data using the same breed classification used in the previous study that examined dogs’ communicative abilities in a relatively large number of breeds [29].

## Materials and Methods

### Ethical Statement

The current study was conducted in strict accordance with the ‘Guidelines for the Treatment of Animals in Behavioural Research and Teaching’ by the Animal Behavior Society/Association for the Study of Animal Behaviour, and was approved by the ethical committee at the Wildlife Research Center, Kyoto University (WRC2010EC001). Dogs were recruited through advertisements in veterinary clinics, trimming salons, local parks and breed specialists. Signed informed consent for participation in this study was obtained from the owners.

### Subjects

All subjects were purebred dogs living as companion animals at their owner’s home. Highly trained dogs (i.e., dogs that engage in sport activity with their owners such as agility, disc, and other games and/or dogs that have training experience for working purpose) were not included in this study. A total of 125 adult dogs participated in this study. Subjects comprised 60 females and 65 males from 26 different breeds. Five dogs (1 Border Collie, 1 Doberman, 1 Portuguese Water Dog, and 2 Shiba Inu) were excluded from the analysis because they were not able to complete the behavioral experiments: two dogs did not take the food rewards from the experimenter’s hand, and the other three never approached the apparatus used in one of the experiments. As a result, 120 dogs consisting of 57 females and 63 males with a mean age of 68.26 months (5.67 years old) were included in this study (Table 1).

**Table 1:** Breed and breed group sample size (*N*), sex (F: female, M: male), and mean age (years) of dogs tested in the present study.

Breed Group	Breed	*N*	Sex	Age
Ancient	Afghan Hound	4	F = 2, M = 2	6.79
(*N* = 24)	Akita Inu	8	F = 5, M = 3	3.47
	Saluki	2	F = 2	2.50
	Shiba Inu	5	F = 3, M = 2	6.83
	Siberian Husky	5	F = 4, M = 1	6.53
Herding	Border Collie	12	F = 3, M = 9	6.90
(*N* = 23)	Welsh Corgi	10	F = 5, M = 5	8.50
	Australian Shepherd	1	M	7.33
Hound	Beagle	5	F = 3, M = 2	8.12
(*N* = 22)	Borzoi	4	F = 3, M = 1	2.58
	Dachshund	6	F = 3, M = 3	9.05
	Irish Wolfhound	1	M	1.33
	Italian Greyhound	6	F = 2, M = 4	6.13
Retriever-Mastiff	Bernese Mountain Dog	5	F = 4, M = 1	5.08
(*N* = 24)	Flat-coated Retriever	2	F = 2	4.42
	Golden Retriever	6	F = 3, M = 3	5.51
	Labrador Retriever	8	F = 6, M = 2	6.92
	Mastiff	1	M	1.58
	Newfoundland	1	M	1.00
	Staffordshire	1	M	1.08
Working	Doberman	5	F = 2, M = 3	6.35
(*N* = 27)	German Shepherd	5	F = 3, M = 2	4.50
	Standard Poodle	5	F = 2, M = 3	2.62
	Toy Poodle	11	F = 2, M = 9	4.72
	Portuguese Water Dog	1	F	2.83

Based on the recently published data on the genetic clustering of dog breeds [21], subjects were classified into five breed groups: Ancient, Herding, Hound, Retriever-Mastiff and Working. The Ancient group consisted of five breeds with similar genetic components to gray wolves [1,21,22], and that were originally from outside central Europe (i.e., Middle-East Asia, East Asia or Siberia). The other four breed groups included 21 breeds originally from European countries that differed in their primary use (i.e., working function) as well as genetic relatedness [21].

### Behavioral Experiments

To evaluate breed differences in producing visual signals, we tested the dog’s spontaneous gaze at human faces using two experimental tasks: the visual contact task and the unsolvable task (see S1 and S2 Movies). Small pieces of food (e.g., chipped beef) were used as rewards in both tasks. To test the dog’s motivation for the reward, the experimenter offered the dog one piece of food before and after each task, confirming that all dogs were highly attracted to the reward. Each dog was tested separately in a familiar environment (e.g., in a room (*N* = 115) or garden (*N* = 5) at the owner’s home) with no leash. In all cases, the test was carried out in a restricted area of at least 2 square meters. To counterbalance the effect of order of task, half of the subjects was given the visual contact task first, and the other half of the subjects was given the unsolvable task first. This counterbalancing procedure was conducted for each breed group. The owner was present throughout the experimental session and was instructed not to give any feedback to the dog for any of its responses. All experimental sessions were videotaped.

#### Visual Contact Task

The visual contact task used in this study is a modification of the one used in Study 2 by Jakovcevic *et al*. [34]. The task consisted of two phases lasting 90 seconds each. In the first warm-up phase, the experimenter moved around the test area while calling the dog’s name and making physical contact with the dog in a friendly manner. The dog was off leash and free to moving around the testing area. During this phase, the subject received at random intervals (mean 14.56 seconds) a total of four pieces of food directly from the hand of the experimenter. Food rewards were placed in a container visible to the subject but out of his/her reach. To focus the dog’s attention towards the feeding place, the experimenter stood at the exact same position, i.e., next to the food container, when giving the food rewards to the subject. Importantly, during this warm-up phase, the experimenter avoided any eye contact with the dog.

Right after the 90-second warm-up phase had elapsed, the test phase started. At this point, the experimenter took one last piece of food and gave it to the dog while standing by the food container. Immediately after that, the experimenter stopped moving and initiated eye contact with the dog. The experimenter offered continuous eye contact but the dog was able to move freely and was not forced to make and/or maintain eye contact with the experimenter until the end of the second phase. The dog’s gaze responses during the second phase were subjected to analysis.

#### Unsolvable Task

The ‘unsolvable task’ [16] consisted of six consecutive ‘solvable’ trials (i.e., the dog could reach the food reward) followed by a single ‘unsolvable’ trial. The experimental apparatus comprised a 12 × 20 cm transparent plastic container and a 30 × 30 cm wooden board. After calling the dog’s name, the experimenter set a piece of food at the center of the wooden board and then put the plastic container over it. The bait of the apparatus was visible to the dogs, but out of their reach (i.e., the experimenter held up the apparatus in front of his/her face while baiting it, and prevented the dogs from touching the apparatus). The experimenter then placed the apparatus on the ground so that the subject was able to manipulate it and get the food reward by removing the container. During the solvable trials all dogs learned how to get the food reward from the apparatus. After the sixth solvable trial, the experimenter presented to the dog one unsolvable trial in which the container was fixed to the wooden board in such a way that the dog could not get the food anymore. During the unsolvable trial, both the experimenter and the owner stood quietly behind the dog at a distance of approximately 1.5 m, while the dog (off leash during the whole experimental session) was free to move around the experimental area. The owner was instructed not to respond to any of the dog behaviors except for eye contact. The dog’s behavior was recorded for 60 seconds after the unsolvable trial was presented.

### Analysis

The dog’s behavior in the two experiments was coded based on the subsequent video analysis. Behavioral coding was made on the 0.3-second time scale by two independent observers naïve to the purpose of the study.

For the visual contact task, we measured: (1) duration of the first gazing (i.e., time from the moment the dog turned/lifted its head towards the experimenter for the first time until the moment it turned its head away from him), and (2) total duration of gazing at the experimenter during the 90-second test phase.

For the unsolvable task, we measured three behavioral variables: (1) latency to the first gazing (i.e., the time elapsed from the moment the unsolvable trial started to the moment the dog turned/lifted its head for the first time back towards the experimenter or the owner), (2) total duration of gazing at the person, and (3) total duration of physical contact with the apparatus (i.e., the time the dog spent manipulating the apparatus including touching, scratching, pushing, sniffing and licking). To evaluate the general tendency of the dog’s gaze responses towards humans, gazing at the experimenter and gazing at the owner were combined.

A subset of the videos (*N* = 30; 25.0%) was randomly selected and coded by an observer naïve to the purpose of the study. Inter-observer reliability testing using Cohen’s Kappa indicated a strong agreement between coders (visual contact task, first gazing duration: *k* = 0.691, *p* < 0.001; total gazing duration: *k* = 0.935, *p* < 0.001; unsolvable task, latency to the first gazing: *k* = 0.760, *p* < 0.001; total gazing duration: *k* = 0.862, *p* < 0.001; total duration of apparatus manipulation: *k* = 0.715, *p* < 0.001).

To examine the effect of breed group on the dog’s gaze responses, we used generalized linear models (GLM). The explanatory variables were breed group (Ancient, Herding, Hound, Retriever-Mastiff or Working), age, their interaction, and sex, while the response variables comprised each of the five behavioral variables. According to the distribution of the response variables, we applied the negative binomial error structure with log link function for the five behavioral variables. ‘Ancient’ and ‘female’ were entered as reference categories when constructing the parameter estimates (*ß*) using GLM. To test the fixed effect of each explanatory variable, the likelihood ratio test with chi-square statistics was carried out (type III test). We used the Steel-Dwass test as a supplementary *post-hoc* test. Effect of age was estimated by calculating Spearman’s ρ or Pearson’s *r*. Analyses were run on R version 2.15.2. (R foundation for Statistical Computing).

## Results

### Visual Contact Task

During the test phase, all dogs except for one male Siberian Husky made eye contact with the experimenter at least once. None of the explanatory variables (i.e., breed group, *ß* = -0.09 [vs. Hound] to 0.60 [vs. Working]; age, *ß* = 0.00; breed group x age, *ß* = 0.00 [vs. Retrievers] to 0.01 [vs. Herding]; sex, *ß* = 0.04, *P* > 0.05) have a significant effect on duration on first gaze (Tables 2 and 3). We found a significant age-effect on the total duration of gazing behavior (*ß* = 0.01, *P* = 0.047), with older dogs gazing for longer than younger ones (Spearman’s *ρ* = 0.289, *P* = 0.001) (Tables 2 and 3). However, breed group (*ß* = 0.56 [vs. Retrievers] to 0.85 [vs. Hound], *P* = 0.212), breed group x age interaction (*ß* = -0.01 [vs. Hound] to -0.00 [vs. Working], *P* = 0.582), and dog’s sex (*ß* = -0.00, *P* = 0.215) did not have any significant effect on total gazing duration (Tables 2 and 3).

**Table 2:** Mean (in seconds) of the behavioral variables according to the different breed groups. Standard deviation is shown in parentheses.

		Ancient	Herding	Hound	Retriever	Working
Visual contact task						
	The first gazing	6.98	11.70	10.74	9.49	14.90
		(10.22)	(12.81)	(12.24)	(8.76)	(13.24)
	Total gazing	34.16	54.59	46.35	47.20	50.79
		(29.42)	(18.23)	(26.97)	(21.56)	(27.94)
Unsolvable task						
	Latency of the first gazing	29.90	18.96	17.41	12.52	14.86
		(19.62)	(15.55)	(15.94)	(12.02)	(16.23)
	Total gazing	4.28	12.80	13.59	17.12	14.90
		(6.52)	(8.48)	(12.25)	(13.35)	(13.59)
	Contact with apparatus	36.71	33.27	29.98	22.98	30.87
		(19.50)	(15.74)	(17.87)	(16.15)	(18.64)

**Table 3:** Results of the GLMs showing the effect of each explanatory variable (i.e., breed group, age, breed group x age, and sex) on dog’s communicative behaviors. Significant results (*p* < 0.05) are shown in bold.

Response variables		Explanatory variables	*df*	Deviance	*P*
Visual contact task					
	The first gazing	Breed group	4	2.13	0.712
		Age	1	0.12	0.734
		Breed group * Age	4	0.74	0.947
		Sex	1	0.05	0.820
	Total gazing	Breed group	4	5.84	0.211
		**Age**	**1**	**3.94**	**0.047**
		Breed group * Age	4	2.85	0.582
		Sex	1	1.54	0.215
Unsolvable task					
	Latency of the first gazing	**Breed group**	**4**	**21.42**	**< 0.001**
		Age	1	0.57	0.449
		Breed group * Age	4	8.89	0.064
		Sex	1	0.38	0.538
	Total gazing	**Breed group**	**4**	**12.42**	**0.014**
		Age	1	0.35	0.555
		Breed group * Age	4	3.53	0.474
		Sex	1	0.09	0.767
	Contact with apparatus	Breed group	4	1.66	0.798
		Age	1	1.12	0.290
		Breed group * Age	4	1.91	0.753
		Sex	1	0.01	0.933

### Unsolvable Task

**Fig 1:**
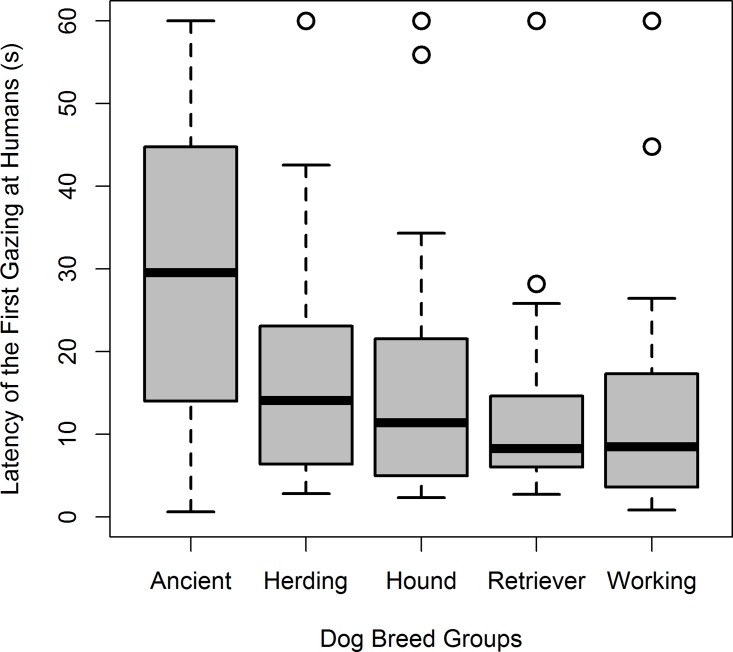
Mean latency (in seconds) to the dog’s first gazing behavior towards humans in the unsolvable task according to dog breed groups. Box-plot depicts subject’s minimum and maximum (whiskers) excluding outliers (blank circles), lower and upper quartiles (edge of the boxes), and medians (line across the box).

During the unsolvable trial, 4.2% of the subjects (two Akita Inu, one Border Collie, one Borzoi, one Labrador Retriever and one Toy Poodle) never looked at the experimenter or the owner. We found a significant difference in the latency to the first gaze according to breed group (*ß* = -0.64 [vs. Hound] to -1.49 [vs. Working], *P* = 0.000) with Ancient breeds gazing later than Retrievers (Steel-Dwass test, *P* = 0.012) and Working breeds (Steel-Dwass test, *P* = 0.033) (Tables 2 and 3, Fig 1). Dog’s sex (*ß* = -0.10, *P* = 0.378), age (*ß* = -0.00, *P* = 0.449), and the interaction of age x breed group (*ß* = 0.00 [vs. Hound] to 0.01 [vs. Working], *P* = 0.064) did not have any significant effect on the latency to the first gaze (Table 3).

**Fig 2:**
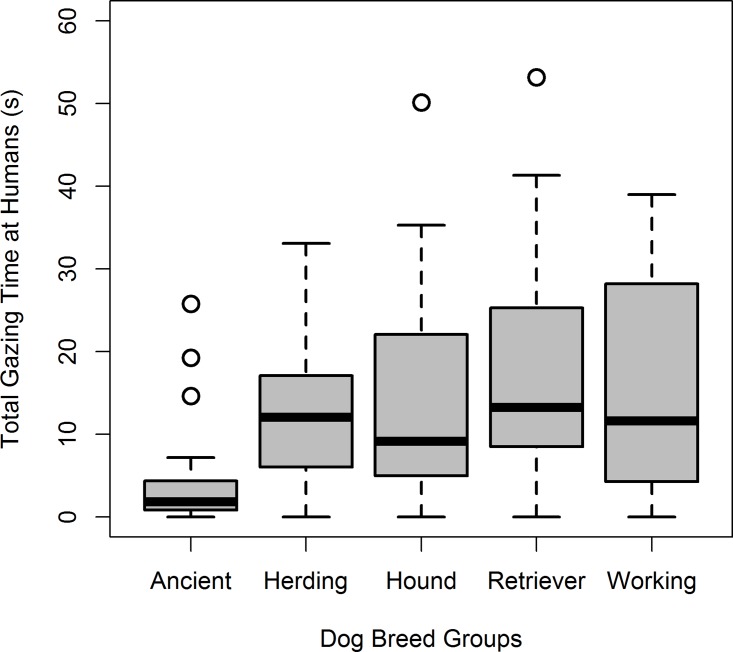
Mean total duration (in seconds) of dog’s gazing behavior towards humans in the unsolvable task according to dog breed groups. Box-plot depicts subject’s minimum and maximum (whiskers) excluding outliers (blank circles), lower and upper quartiles (edge of the boxes), and medians (line across the box).

For the total duration of gazing, we found a significant effect of breed group (*ß* = 1.12 [vs. Hound] to 1.85 [vs. Working], *P* = 0.015) with Ancient breeds gazing for shorter periods of time than Herding (Steel-Dwass test, *P* = 0.002), Hound (Steel-Dwass test, *P* = 0.002), Retrievers (Steel-Dwass test, *P* < 0.001) and Working breeds (Steel-Dwass test, *P* = 0.011) (Tables 2 and 3, Fig 2). However, dog’s sex (*ß* = -0.05, *P* = 0.766), age (*ß* = 0.00, *P* = 0.555), and the interaction of age x breed group (*ß* = -0.01 [vs. Working] to -0.00 [vs. Hound], *P* = 0.474) did not have any significant effect (Table 3).

Finally, the mean duration of physical contact with the apparatus was not affected by any of the three explanatory variables included in the analysis (i.e., breed group, *ß* = -0.31 [vs. Retrievers] to 0.03 [vs. Hound]; age, *ß* = 0.00; breed group x age, *ß* = -0.00 [vs. Hound] to 0.00 [vs. Working]; sex, *ß* = 0.01, *P* > 0.05, Tables 2 and 3).

### Comparison with a previous study

We then followed the classification of Passalacqua *et al*. [29] and re-ran the analysis using only the dog breeds that were used in their study (Primitive [Akita Inu, Shiba Inu, Siberian Husky]: *N* = 19, Hunting/herding [Australian Shepherd, Beagle, Border Collie, Duchshund, Flat-coated Retriever, Golden Retriever, Labrador Retriever,]: *N* = 40, Molossoid [Bernese Mountain Dog, Staffordshire, German Shepherd]: *N* = 11). Nevertheless, the results were in accordance with our findings that Ancient breeds gazed at humans for a shorter period of time (see S1 Table. Visual contact task: Steel-Dwass test, Total gaze duration: Primitive vs. Hunting/Herding, *P* = 0.033, Primitive vs. Molossoid, *P* = 0.830, Hunting/Herding vs. Molossoid, *P* = 0.342; Unsolvable task: Steel-Dwass test, Latency of the first gaze: Primitive vs. Hunting/Herding, *P* = 0.008, Primitive vs. Molossoid, *P* = 0.044, Hunting/Herding vs. Molossoid, *P* = 0.949; Total gaze duration: Primitive vs. Hunting/Herding, *P* < 0.001, Primitive vs. Molossoid, *P* = 0.102, Hunting/Herding vs. Molossoid, *P* = 0.702). Furthermore, in contrast to Passalacqua *et al*. [29], there was no significant difference between Hunting/Herding and Molossoid breeds in any communicative behavior in this additional analysis.

## Discussion

To estimate the influence of selective breeding on modern dog’s ability to exchange visual communicative signals with humans, the present study examined potential breed group differences in human-directed gazing behavior using two behavioral tasks. During the tests, almost all dogs gazed spontaneously at humans suggesting that modern domestic dogs frequently send visual signals to humans as communicative cues when seeking food rewards [16,36].

However, not all breeds were equally prone to using these social cues. We found that it took longer for Ancient breed dogs to establish eye contact with humans, and that they gazed at human faces for shorter periods of time than other breed groups in the unsolvable situation. It could be argued that these inter-breed differences are merely the result of inter-individual differences in motivation for seeking food rewards and/or the dog’s persistency to engage in a problem-solving task [37,38]. However, this is unlikely since breed differences were not found in the duration of physical manipulation of the apparatus during the unsolvable task, and all dogs consumed the piece of food offered by the experimenter at the end of the experimental sessions. Thus, these results suggest that the level of engagement in the unsolvable task did not differ according to breed groups, but that Ancient breeds were particularly less prone to use gaze signals with humans even though they are equally motivated to seek the reward.

To explore situation-dependency of behavioral patterns among the different breed groups, we used multiple behavioral tasks. Results indicated that a statistically significant effect of breed group was found only in the unsolvable situation. Thus, dog breed differences in human-directed gazing behavior seem to vary depending on task or situation. In the visual contact task human’s gaze preceded dog’s gaze, and the dogs had only to keep eye-contact with humans for begging for the reward. On the other hand, in the unsolvable task the dogs had to divert their attention from the experimental apparatus and spontaneously produce gazing behavior–turn back and look at the humans–in an attempt to send them communicative signals. The latter could be considered more complex due to the involvement of a problem-solving component, and the maintenance of the dog’s gaze was lower than in the former (i.e., the allocation of time in human-directed gazing was shorter in the unsolvable task [20.96%] than in the visual contact task [51.85%]; Steel-Dwass test, *P* < 0.001). It is possible that, regardless of breed group difference, dogs have commonly developed an ability to maintain eye-contact with humans in response to human-given gaze, whereas dog’s ability for spontaneously producing gazing behavior towards humans has been partially influenced by genetic factors associated with breed clustering.

Previous studies using the ‘unsolvable task’ yielded similar results when analyzing species or breed differences in the use of gaze signals towards humans. Miklósi *et al*. [16] found that wolves showed a longer latency to the first gazing behavior, and a shorter duration of total gazing towards humans compared to domestic dogs. Passalacqua *et al*. [29] examined breed difference in human-directed gazing behavior and reported that Primitive breeds, which were comparable to Ancient breeds used in this study, gazed at humans for shorter periods of time than Hunting/Herding breeds, although the total duration of gazing behavior did not differ between Primitive and Molossoid breed groups. Together with our results on Ancient breeds, these findings support the ‘wolf-remnant’ hypothesis since both non-domesticated canine species and Ancient dog breeds are less likely to produce spontaneous gaze signals towards humans.

Recent genomic studies of modern purebred dogs have identified major breed clusters distinguishing dogs with similar genetic signatures to wolves (i.e., Ancient breeds) from those under more recent intense artificial selection [21,22]. In the present study, we found a clear behavioral distinction between Ancient breeds and other breed groups, which corresponds to a larger genetic distance between them. Given that dogs of the Ancient breed group are diverse in geographical origin, morphology and working purpose [1], it is likely that a genetic component shared among those breeds (i.e., genetic similarity with wolves) may have a significant impact on dog’s human-directed gazing behavior. Thus, that Ancient breeds engaged in less gazing behavior suggests that a dog’s communicative ability to convey visual signals to humans may be linked to their genetic similarity to wolves, providing further support to the ‘wolf remnant’ hypothesis.

The idea that canine behavior has been significantly altered by divergence between wolf-like Ancient breeds and other modern primary breeds is also supported by several sources of published data on the sensitivity of dogs to human-given social cues. Studies on wild dog breeds, which have experienced less of artificial selection, have shown that although dingoes and New Guinea singing dogs are able to respond to human social cues they seem to be less sensitive than other domestic dogs [30,31]. Therefore, it seems plausible that a dog’s predisposition for communicating with humans has been enhanced by the artificial selection involved in the creation of modern European breeds [30,31].

However, other studies of breed differences have reported a greater influence of selective breeding on a dog’s social cognitive skills involved in specific ‘cooperative’ work with humans such as retrieving prey, hunting with human partners and herding or guarding sheep [28–30,34]. This ‘working purpose’ hypothesis is supported by the findings of the study by Passalacqua *et al*. [29] in which the Hunting/Herding group was found to look towards humans for longer times than the Primitive or the Molossoid breeds. In contrast, the current study showed no clear differences within the different types of working groups (Herding, Hunting, Retriever-Mastiff and Working). The discrepancy among the studies could be due to the different categories of breeds used in each study. Perhaps the inclusion of different breeds may have lead to the discrepant results. For instance, the three mastiff-type breeds (Boxer, Bull Terrier, and Rottweiler) that were included and classified as Molossoid breeds in Passalacqua *et al*. [29] were not present in our study.

It is possible that the complicated breeding history of modern purebred dogs makes it hard to detect any clear genetic or behavioral signature created during the selection process for particular working purposes. In fact, researchers are faced with the difficult challenge of estimating a single original purpose along with the resulting selective force for each breed [20]. For instance, the German Shepherd is thought to be originally bred for herding and guarding livestock but subsequently has also been used for search and rescue, as well as for police and military roles [24,25]. Moreover, the current breeding of show dogs and companion dogs may be also associated with modifying behavioral traits in purebred dogs, an idea that has recently received support from a study on dog’s personalities [39]. If this is the case, then lineage differences within a single breed could also lead to behavioral differences. Since modern purebred dogs have been established through various selective pressures at different points during their breeding history, the domestication of dogs can be considered to be still in progress [20,39]. Further investigations focusing on a more detailed analysis of breeding processes is warranted to elucidate the influence of a specific selective pressure on canine behavior. For instance, with the further progress in canine genomic study, it would be important that future studies take into account the actual genetic distance of each particular breed from the wolf.

Our results also show an effect of age on the use of visual signals towards humans, which may reflect the effect of a dog’s prior experiences on communication with humans. The present study tested only adult dogs (more than 12 months old), with the assumption that their behavior has already been fully formed by social experience through everyday interaction with their human partners. We found that older dogs gazed for longer times at the experimenter in the visual contact task. This result may be in line with previous findings showing that dog’s performance is associated with living conditions and early experiences [40–42]. For instance, household dogs gaze at humans for longer than shelter-housed dogs in a similar visual contact situation [41]. Moreover, while dog’s performance utilizing human gestural cues to locate hidden food appears at an early age and does not improve with developmental changes [15], dog’s use of gazing behavior towards humans greatly improves with age [29]. Although all subjects were household pet dogs that had not received any professional training, we cannot rule out the possibility that differences in everyday interaction with their owners and/or previous experience in requesting help from humans could have accounted for part of the observed variability. In fact, it is likely that the ability to interact with humans has been shaped by a complex interaction between the breed’s inherited character and the individual dog’s experience during ontogeny [7,29,43]. Further research should try to evaluate the degree to which prior experience in similar scenarios (e.g., how much they beg for food while their owners are eating) is relevant, and incorporate that measure into the analyses.

In conclusion, the present study shows that dog breed difference in human-directed gazing behavior between Ancient breeds and other breed groups is much larger than those among non-Ancient purebred breeds. This pattern is particularly apparent in the unsolvable situation, with Ancient breeds less prone to sending spontaneous gaze signals towards humans than other European breeds. Our findings suggest that cross-specific communicative ability is acquired during an earlier split between wolf-like Ancient breeds and other primary breeds, although it might have been enhanced over the course of breed creation, which continues up to the present day.

## Supporting Information

S1 Movie A video example of visual contact task. (MP4)

S2 Movie A video example of unsolvable task. (MP4)

S1 Table Results of the GLMs showing the effect of each explanatory variable (i.e., breed group [Primitive, Hunting/Herding, Molossoid], sex, and age) on dog’s communicative behaviors. Breed group are categorized according to Passalacqua *et al*. [29]. Significant results (*p* < 0.05) are shown in bold.
(DOCX)
