# Supplementary material for: Dog Breed Differences in Visual Communication with Humans
Source: PLoS One. 2016 Oct 13;11(10):e0164760. doi: 10.1371/journal.pone.0164760 (PMC5063391; doi:10.1371/journal.pone.0164760)
Supplement: S1 Table — Breed group are categorized according to Passalacqua et al. [29]. Significant results (p < 0.05) are shown in bold. (DOCX) [file pone.0164760.s003.docx]

S1Table. Results of the GLMs showing the effect of each explanatory variable (i.e., breed group [Primitive, Hunting/Herding, Molossoid], sex, and age) on dog’s communicative behaviors. Breed group are categorized according to Passalacqua *et al.* [29]. Significant results (*p* < 0.05) are shown in bold.

| Response variables | | Explanatory variables | *df* | Deviance | *P* |
| --- | --- | --- | --- | --- | --- |
| Visual contact task | |  |  |  |  |
|  | The first gazing | Breed group | 2 | 3.84 | 0.146 |
|  |  | Age | 1 | 2.69 | 0.101 |
|  |  | Breed group * Age | 2 | 2.11 | 0.349 |
|  |  | Sex | 1 | 0.02 | 0.884 |
|  | Total gazing | Breed group | 2 | 5.25 | 0.072 |
|  |  | Age | 1 | 0.61 | 0.433 |
|  |  | Breed group * Age | 2 | 2.13 | 0.344 |
|  |  | Sex | 1 | 0.58 | 0.446 |
| Unsolvable task | |  |  |  |  |
|  | Latency of the first gazing | **Breed group** | **2** | **15.08** | **< 0.001** |
|  |  | **Age** | **1** | **9.68** | **0.002** |
|  |  | **Breed group * Age** | **2** | **8.89** | **0.017** |
|  |  | Sex | 1 | 0.46 | 0.830 |
|  | Total gazing | **Breed group** | **2** | **7.92** | **0.019** |
|  |  | Age | 1 | 0.07 | 0.784 |
|  |  | Breed group * Age | 2 | 1.95 | 0.378 |
|  |  | Sex | 1 | 0.05 | 0.815 |
|  | Contact with apparatus | Breed group | 2 | 0.51 | 0.775 |
|  |  | Age | 1 | 1.41 | 0.235 |
|  |  | Breed group * Age | 2 | 0.05 | 0.975 |
|  |  | Sex | 1 | 0.06 | 0.799 |
